# Personalizing Colon Cancer Therapeutics: Targeting Old and New Mechanisms of Action

**DOI:** 10.3390/ph6080988

**Published:** 2013-08-21

**Authors:** Christina Leah B. Kline, Wafik S. El-Deiry

**Affiliations:** Hematology/Oncology Division, Penn State Hershey Medical Center, Hershey, PA 17033, USA; E-Mail: ckline@hmc.psu.edu

**Keywords:** colorectal cancer, personalized medicine, genetic polymorphisms

## Abstract

The use of pharmaceuticals for colon cancer treatment has been increasingly personalized, in part due to the development of new molecular tools. In this review, we discuss the old and new colon cancer chemotherapeutics, and the parameters that have been shown to be predictive of efficacy and safety of these chemotherapeutics. In addition, we discuss how alternate pharmaceuticals have been developed in light of a potential lack of response or resistance to a particular chemotherapeutic.

## 1. Introduction: Personalizing Colon Cancer Therapeutics

Personalized medicine is at the heart of patient care as doctors adjust therapeutic strategies based on their personal observations of their patients. With the development of 21st century scientific tools, however, personalized medicine has been taken to a different level. The benefits of these are now being increasingly seen in the treatment of patients with colon cancer.

It is imperative that we afford the advantages of personalized cancer therapy to colon cancer patients. Colon cancer is the 4th leading cause of cancer-related death worldwide [1]. The rate of survival for colon cancer depends largely on the pathologic state of the disease. Currently, the treatment strategy is also highly dependent on the stage of the disease at diagnosis. In disease stages where cancer cells have not spread to distant organs (Stages II and III), surgical removal of the tumor is warranted. In Stage III tumors, the stage where the cancer cells have spread to lymph nodes, chemotherapeutics are also given after surgery. This is what is referred to as adjuvant chemotherapy. It has been shown that giving adjuvant chemotherapy is superior to surgical intervention alone in terms of preventing disease recurrence [2] and improving disease-free and overall survival [3]. The 5-year survival rate for Stage III cancer patients is 69.2%. Once the colon cancer cells have spread to other organs in the case of metastatic colon cancer, the 5-year survival rate of patients drops drastically to 11.7% [4]. Thus, much research is being done to develop therapeutic strategies for metastatic colon cancer. In addition to the need to increase the efficacy of current chemotherapeutic regimens, there is also a need to reduce toxicity. Eighty percent of patients undergoing chemotherapy experience side-effects [5]. These side effects are brought about at least in part by the effects of the chemotherapeutics on normal cells. These efficacy and toxicity data underscore the need for more effective and targeted therapeutic regimen.

In this review, we discuss how the use of old and new colon cancer chemotherapeutic drugs is increasingly being personalized to maximize the benefit to the patients. In addition, we discuss how alternate pharmaceuticals have been developed in light of a potential lack of response or resistance to a particular chemotherapeutic.

## 2. Targeting Old Mechanisms of Action: Inhibitors that Target the DNA

### 2.1. Personalizing the Use of a 55-Year Old Drug: 5-Fluorouracil (5-FU)

#### 2.1.1. How 5-FU Works

**Figure 1:**
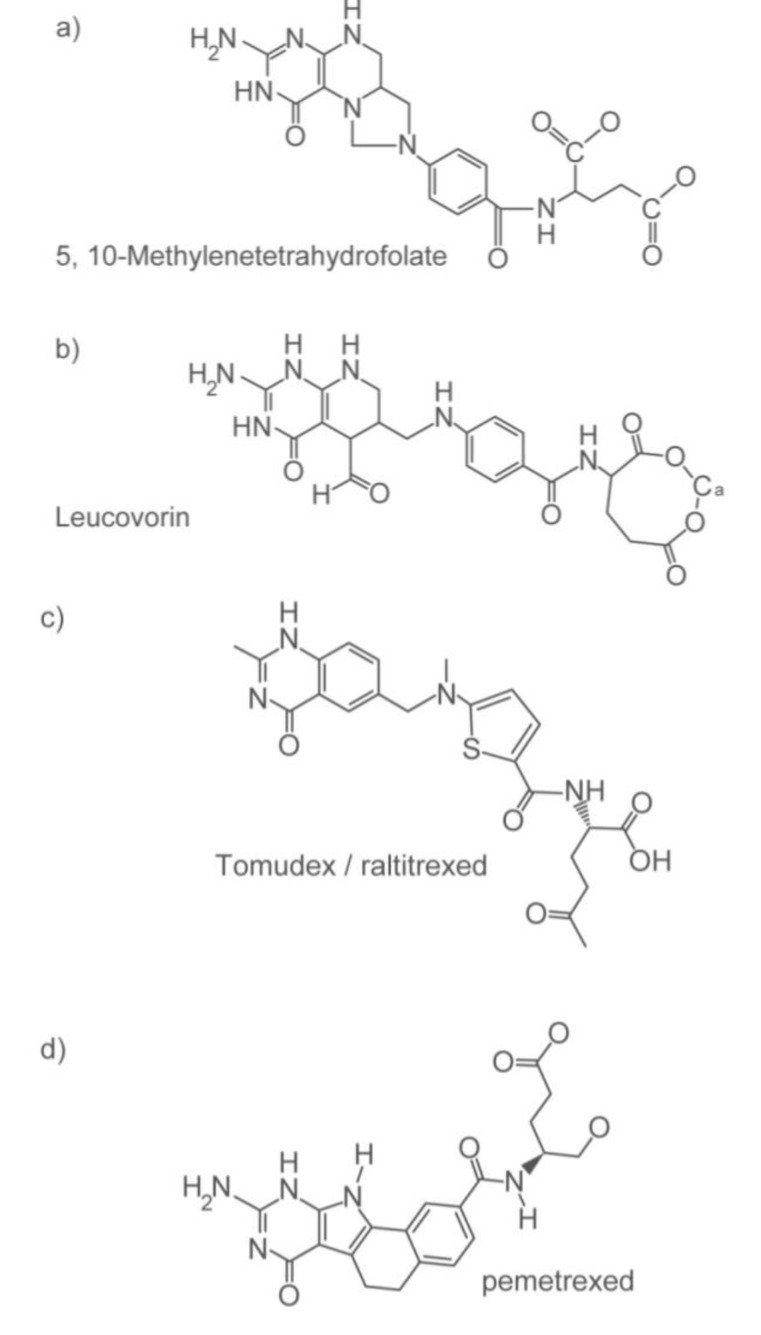
Chemical structures of (**a**) 5,10-methylene-THF, (**b**) leucovorin and the antifolate inhibitors (**c**) tomudex and (**d**) pemetrexed.

5-FU has been the mainstay of colon cancer chemotherapy for more than 50 years. This is probably in part due to its impact on multiple pathways—DNA synthesis, DNA repair, and RNA metabolism (Figure 1). After promising results with tumors grown in rats and mice [6], 5-FU was tried in humans, first, by giving a single intravenous injection for 5 days. Unfortunately, tumor regression was only observed when toxic 5-FU concentrations were used. Thus, for decades, studies have been done to optimize the dose [7], schedule [8,9,10] and mode of administration [11,12] to maximize therapeutic effect and minimize toxicity. Currently, 5-FU is administered with leucovorin in regimens that consist of combinations of bolus injection and continuous intravenous infusions.

**Scheme 1:**
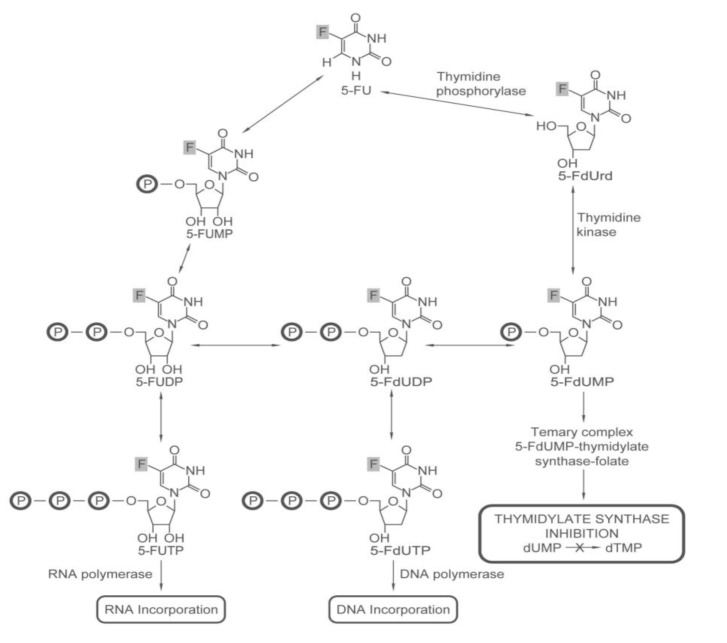
Anabolism and effects of 5-FU. 5-FU can be metabolized in cells via different pathways.

**Scheme 2:**
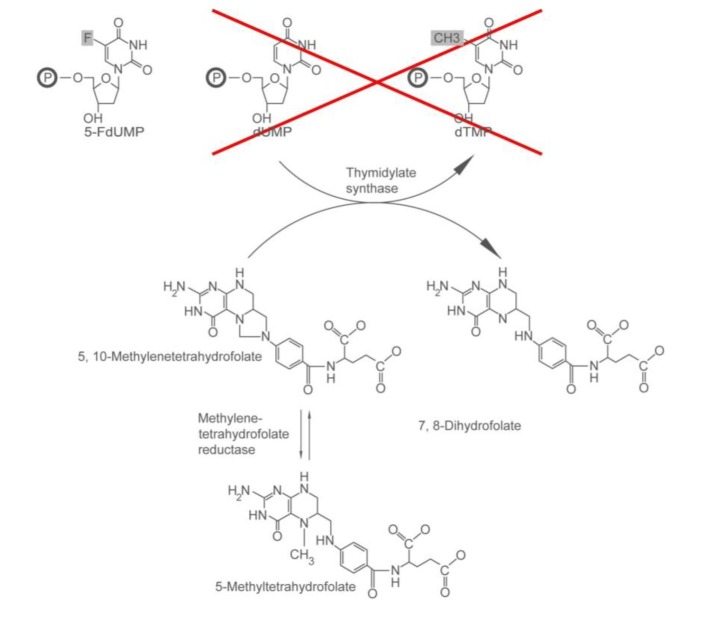
Activities of thymidylate synthase and methylene tetrahydrofolate reductase. Thymidylate synthase converts dUTP to dTMP, in the presence of 5,10-methylene-THF. 5-FdUMP competes with dUMP, blocking dTMP synthesis. Methylene tetrahydrofolate reductase reduces 5, 10-methylene-THF levels by converting it to 5-methyltetrahydrofolate.

The development of 5-FU as a chemotherapeutic came, in part, from the seminal observation that there was enhanced utilization of uracil by rat liver tumor cells in comparison to their normal liver cell counterparts [13]. Both uracil and 5-fluorouracil can be metabolized in cells to form nucleoside monophosphates (Scheme 1). Their nucleoside monophosphate derivatives can complex with thymidylate synthase in the presence of the cofactor 5,10-methylenetetrahydrofolate (5,10-methylene-THF) (Scheme 2). In the case of the 5-fluoro-2'-deoxyuridine monophosphate formed from 5-FU, however, the carbon-fluorine bond cannot be cleaved by thymidylate synthase. This locks the enzyme in the complex, making it less available for deoxythymidine monophosphate (dTMP) synthesis [14]. Consequently, DNA replication is inhibited. The conversion of 5-FU to its nucleotide monophosphate form is catalyzed by thymidine phosphorylase and thymidine kinase. Thymidine phosphorylase expression [15] and activity [16] have been found to be significantly higher in tumor tissue than in noncancerous tissue. This can explain in part why 5-FU hits tumor cells harder. On the other hand, because 5-FU targets DNA synthesis, actively dividing normal cells (e.g., cells lining the gut, the skin, blood cells) are also affected. Thus, gastrointestinal toxicity, bone marrow suppression and hand-foot syndrome are side-effects of 5-FU based chemotherapy [17].

The impact of 5-FU goes beyond the downregulation of dTMP synthesis. As a result of thymidylate synthase inhibition by 5-FU, deoxyuridine monophosphate (dUMP) and deoxyuridine triphosphate (dUTP) accumulate [18,19,20,21]. dUTP accumulation promotes the misincorporation of uracil into DNA by DNA polymerase. Although the DNA repair pathway can excise the misincorporated uracil [22,23], the excised dUMP can be rephosphorylated to dUTP and reincorporated and the process keeps on repeating in what is referred to as futile cycling. DNA strand breakage [24] and lethal damage occurs as the repair mechanism fails.

Fluorouridine triphosphate (FUTP) formed from 5-FU disrupts RNA metabolism. FUTP can be incorporated into the 45S and 32S pre-ribosomal RNA, inhibiting the processing of these molecules into the 28S and 18S RNA components of the ribosome [25]. Pre-mRNA splicing is inhibited as a result of insertion of FUTP into the U2 spliceosomal snRNA. 5-FU, therefore, disrupts key metabolic processes involving RNA; namely, pre-mRNA splicing, ribosome formation, and protein synthesis.

The response rate to 5-FU monotherapy among colorectal cancer patients is 11% [26]. Thus, much research has been done to increase the efficacy of 5-FU. Given that one of the targets of 5-FU is thymidylate synthase (TS), efforts to increase the duration of the thymidylate synthase inhibition have been made. A strategy to accomplish this is to increase the levels of the cofactor 5,10-methylene-THF. Increased 5,10-methylene-THF levels promote the maximal binding of FdUMP to TS [27] and decrease the dissociation of the FdUMP-TS complex [28].

Folinic acid or leucovorin (LV), a formyl derivative of 5,10-CH2-THF, can be converted to 5,10-methylene-THF in cells, increasing the efficacy of 5-FU and FdUMP [27,29]. Given the favorable *in vitro* effects of leucovorin in combination with 5-FU, it has been administered with 5-FU in the clinic. The 5-FU/LV combination increased response rate to 23% [26], and prolonged overall survival and time to disease progression [30,31].

#### 2.1.2. Who May and May not Benefit from 5-FU

Currently, whatever stage of the disease a colorectal cancer patient has, the first option that will be offered to them for chemotherapy would involve 5-FU as a single agent or in combination with other drugs, depending on the stage of the tumor and the patient’s performance status. It is therefore beneficial to be able to identify the factors that determine 5-FU response. Efforts are being made to identify mutations or genetic polymorphisms in patients who are sensitive or resistant to 5-FU. As has been mentioned above, one of the targets of 5-FU is the enzyme thymidylate synthase. The therapeutic response to 5-FU has been shown to be associated with the extent of inhibition of thymidylate synthase activity [32]. The extent of inhibition of TS activity depend in part on the levels of thymidylate synthase and 5,10-methylene-THF. The amount of 5,10-methyleneTHF in cells is regulated by methylenetetrahydrofolate reductase (MTHFR). MTHFR reduces the cofactor to 5-methyltetrahydrofolate [33] (Scheme 2). Given the importance of the level of TS and the activity of MTHFR to thymidylate synthase inhibition, candidate genes that would influence response to 5-FU would be the genes that encode these two enzymes, *TYMS* (for TS) and *MTHFR* (for MTHFR). A common variant of *MTHFR* is 677C>T (A222V). This polymorphism has been associated with reduced MTHFR activity [34]. In a number of studies [35,36], the presence of the polymorphism, especially in a homozygous fashion [37,38], has been correlated with response to 5-FU-based treatment. In other studies, no relationship has been found between the polymorphism and response to different 5-FU based chemotherapeutic regimens [39,40]. The conflicting results on the impact of *MTHFR* polymorphism on response to 5-FU can explain at least in part why screening for *MTHFR* variant prior to 5-FU-based treatment has not been adopted. *TYMS* polymorphisms have also been shown to influence sensitivity to 5-FU-based chemotherapy. The promoter enhancer region of the *TYMS* gene contains a tandemly repeated sequence [41]. The number of repeats of this sequence influences the translational efficiency of the *TYMS* mRNA [42,43]. The most common alleles have a double repeat (*2R*) and triple repeat (*3R*) [44]. Cells [42] and gastrointestinal cancer tissues [45] that have 3 repeats (*3R*) of this sequence have higher TS expression [46]. Poorer response to 5-FU has been seen among patients with the *3R* allele [46,47,48,49]. Not all patients, however, that are *3R/3R* have high TS expression [46] or are unresponsive to 5FU treatment [50]. A G>C variant has been identified in the 2nd repeat of the *3R* allele [51]. This transversion reduces the transcriptional activity of the *TYMS* promoter. Patients that homozygous *3R* but have the G>C variant (*3C) have lower TS expression and respond better to 5-FU than patients with the *3R* allele containing the G (3G) [52].

The use of thymidylate synthase expression in resected tumors to predict 5-FU response has been explored. Patients that have high *TYMS* expression levels in their resected tumors respond poorly to 5-FU and leucovorin [53,54,55,56] and to a combination of 5-FU/LV and oxaliplatin [57]. On the other hand, not all patients that have low *TYMS* expression respond to 5-FU-based therapy [54]. Thus, TS expression may actually be more of a predictor of 5-FU resistance, rather than response.

*TYMS* copy number gains may also be predictive of 5-FU resistance. Patients that have been previously treated with 5-FU based chemotherapy and had amplification of their *TYMS* gene had shorter survival times than patients that did not have *TYMS* gene amplification [58,59].

The efficacy of 5-FU-based chemotherapy is dependent in part on the 5-FU plasma levels achieved in a patient [60,61,62]. Given that 80% of administered 5-FU is degraded [63] and only 1–3% of the drug is actually anabolized [64], the extent of 5-FU catabolism largely determines 5-FU levels. The rate-limiting step in 5-FU catabolism is catalyzed by the enzyme dihydropyrimidine dehydrogenase (DPD) [64]. Low DPD expression has been observed among patients who responded to 5-FU treatment [65,66]. However, no genetic polymorphism has been found to be predictive of DPD deficiency [67]. Nonetheless, some DPYD SNPs have been proposed for clinical use to avoid severe side effects from 5-FU [68] (see below).

One of the mechanisms of action of 5-FU involves the DNA repair pathway. It is conceivable, therefore, that response to 5-FU can be affected by the status of the DNA repair machinery. It has been found that 12–28% of colorectal cancers have numerous mutations at microsatellite sequences—a phenotype that has been referred to as microsatellite instability (MSI) [69]. These mutations are brought about by defects in the DNA mismatch repair (MMR) system. Stage II and stage III patients that have MSI-high tumors [70] or mismatch repair deficiency [71] do not appear to benefit from adjuvant 5-FU based chemotherapy. One mechanism that has been proposed to explain this is the higher incorporation of 5-FU into DNA in the presence of a deficient mismatch repair system, and therefore, reduced formation of the FdUMP-TS complex [72]. In light of the lack of response to 5-FU among patients with MSI-high tumors, MSI testing is now recommended for Stage II patients prior to treating them with single-agent 5-fluorouracil [73]. In the case of Stage III patients, even if those with MSI-high tumors may not respond to 5-FU, these patients are more commonly treated with 5-FU in combination with oxaliplatin, in a regimen referred to as FOLFOX. Stage III patients with deficient mismatch repair have been shown to respond better to FOLFOX than to 5-FU alone [74].

The administration of chemotherapy does not only involve maximizing response but also minimizing toxicity. Close to half of patients who have received 5-FU/leucovorin have experienced toxicities that necessitated dose reduction [75]. 5-FU induced toxicity has been associated with 5-FU plasma levels [76]. As has been mentioned above, 5-FU levels are largely determined by the rate of 5-FU catabolism and the activity of DPD [77]. Patients with DPD deficiency have high 5-FU plasma levels [78,79], and thus experience severe side effects [80]. It is estimated that approximately 2–12% of colorectal cancer patients have at least partial deficiency in DPD [81]. The *DPYD* gene has been implicated the most in 5-FU-induced toxicity. The most common *DPYD* single nucleotide polymorphism (SNP) that has been associated with 5-FU-induced toxicity is the splice-site mutation c.1905+1G>A (IVS14+1G>A) [68,82,83]. Two other SNP’s that have been correlated with adverse side effects are 2846A>T [68,84] and 1679T>G [68]. Sixty percent of patients with either IVS14+1G>A or 2846A>T have experienced severe side effects [68,85]. Aside from a subset of *DPYD* polymorphisms, the combination of an *MTHFR* polymorphism (*MTHFR 1298* A>C) with *TYMS* 3′-UTR ins/del polymorphisms has been associated with 5-FU toxicity [44].

Given the preceding discussion, are we close to identifying patients who will benefit from and patients who will react adversely to 5-FU? Currently, screening for MSI is recommended for patients with Stage II disease prior to administering single-agent 5-FU. If they are found to have MSI-H tumors, treating them with 5-FU may bring more harm than benefit. There may be benefit in screening for *TYMS* polymorphisms, especially the *3R* allele containing the G, to identify patients that are less likely to respond to 5-FU. In addition, it is important to identify patients who are predicted to experience severe adverse side-effects from 5-FU, specifically those with the following *DPYD* polymorphisms: IVS14+1G>A or 2846A>T. Analyzing metastatic lesions of patients previously treated with 5-FU for TYMS gene amplification can guide decisions as to whether to re-treat these patients with 5-FU. Previous results suggest that re-treating patients with TYMS amplification may not provide additional benefit and also expose them unnecessarily to 5-FU’s toxic effects. On the other hand, the use of TS and DPYD expression in tumors as a screening strategy must be done with caution. Exposure to 5-FU itself perturbs the expression of these two enzymes. DPD expression [86] and activity [87] can be downregulated in response to 5-FU treatment. On the other hand, TS expression is upregulated in response to 5-FU treatment *in vitro* [88] and *in vivo* [89]. Another caveat in the use of TS expression is that although low TS has been predictive of response in metastatic colorectal cancer patients, the opposite has been found in patients with Stage II and Stage III disease on adjuvant therapy [90,91,92].

#### 2.1.3. Options for Those Who May not Benefit from 5-FU

All is not lost for patients who are expected to react adversely to 5-FU-based chemotherapy. Toxicities can be avoided in these patients by administering reduced doses of 5-FU [93] and by closely monitoring 5-FU levels during treatment. The latter is referred to as 5-FU pharmacokinetic (PK) monitoring. 5-FU doses have been determined conventionally by calculating body surface area (BSA) from the patient’s weight and height. With 5-FU pharmacokinetic monitoring, however, actual 5-FU levels are measured during administration of continuous infusion 5-FU chemotherapy. 5-FU doses are adjusted in the succeeding cycles of chemotherapy to achieve 5-FU levels that are within what is referred to as the therapeutic range. The minimum value in this range is the minimum 5-FU plasma level that has been observed to be efficacious. The maximum value in this range is the highest 5-FU plasma level that has been observed that has not resulted in dose-limiting toxicities in patients. The therapeutic range is dependent on the mode of administration of 5-FU. The benefits of PK monitoring in metastatic colorectal cancer patients have been shown in a number of studies. In patients treated with 5-FU and leucovorin via a weekly 8-H infusion, objective response rate was found to be increased and toxicity was decreased with the use of PK monitoring versus the use of the BSA method [94]. Similar results were observed when 5-FU/LV was combined with oxaliplatin in the FOLFOX regimen [95]. Through PK monitoring, a patient who is predicted to react adversely to 5-FU by virtue of an IVS+G>A *DPYD* polymorphism can be given 5-FU at a reduced dose [68].

The degree of TS inhibition achieved with 5-FU/LV in patients has been associated with therapeutic response [32]. Thus, the use of TS inhibitors for colorectal cancer therapy continues. In the case of 5-FU, the 5-FU metabolite FdUMP competes against the normal substrate of TS in cells, dUMP. On the other hand, TS inhibitors have been developed to compete against the cofactor of TS, 5,10-methylene-THF. These inhibitors are referred to as antifolate inhibitors (Figure 1). Tomudex (Raltitrexed^®^, Astra Zeneca) is an antifolate TS inhibitor that is now used in clinics outside the USA as an alternative to 5-FU for patients who are sensitive to 5-FU and the 5-FU oral prodrug form, capecitabine, including in a patient with DPD deficiency [96,97,98]. Tomudex has been shown to have similar efficacy as 5-FU in combination with other agents used with 5-FU (e.g., oxaliplatin) [99]. Unfortunately, tomudex can also cause toxic side effects, including death [100,101,102]. The mechanism behind the adverse effects of tomudex, however, is not well elucidated. Pemetrexed (Alimta™, Lilly, first referred to as LY231514), another antifolate TS inhibitor [103], targets two other enzymes involved in folate metabolism—dihydrofolate reductase and glycinamide ribonucleotide formyltransferase. The efficacy and safety of pemetrexed in colorectal cancer therapy, whether as a single-agent [104,105] or in combination with irinotecan [106,107] has been shown to be comparable but nonsuperior to 5-FU. The use of pemetrexed among patients predicted to react adversely to 5-FU remains to be explored [107].

### 2.2. Personalizing the Choice of DNA-Damaging Agent: Oxaliplatin or Irinotecan

#### 2.2.1. How Oxaliplatin and Irinotecan Work

**Scheme 3:**
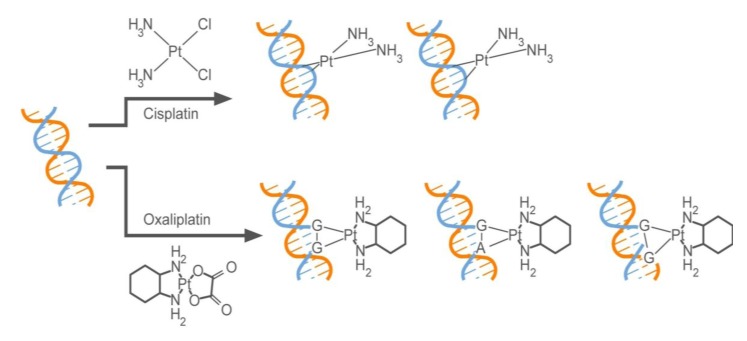
Structures of DNA adducts formed by a) cisplatin and b) oxaliplatin.

Chemotherapeutics have been combined with 5-FU and leucovorin to increase treatment success. The use of platinum compounds for cancer chemotherapy started with the observation that bacterial cell division is inhibited by platinum compounds [108]. Among 34 compounds in this chemical class that have been tested, cisplatin (*cis*-dichlorodiamine platinum II) was shown to have the highest antitumor activity in mice [109]. However, partly due to the toxicity of cisplatin [110], a platinum (II) complex of *trans-l-*diaminocyclohexane, oxaliplatin was synthesized. Oxaliplatin has been shown to be as effective as and less toxic than cisplatin for therapy of colorectal cancer [111], although cisplatin continues to be used for non-colorectal cancer tumor types. Oxaliplatin and cisplatin bind to DNA and form adducts (Scheme 3) [112,113]. The structures of the DNA adducts that they form, however, differ by virtue of the diaminocyclohexane (DACH) ring of oxaliplatin. Mismatch repair proteins recognize the cisplatin-DNA adducts [114,115], resulting in the eventual engagement of apoptosis. However, cancer cells that have a defective mismatch repair system escape this killing mechanism and exhibit resistance to cisplatin [116]. On the other hand, the hydrophobicity and bulkiness of the DACH ring of oxaliplatin prevents the binding of the mismatch repair proteins [117], and therefore, the cytotoxic effect of oxaliplatin is not dependent on a functional mismatch repair system. This can partly explain the observation that cells that have lost a functional mismatch repair system lose their susceptibility to cisplatin but not to oxaliplatin [116]. The addition of oxaliplatin to 5-FU/LV in the clinic in a regimen referred to as FOLFOX [118] has resulted in a 50–60% response rate [119,120] and prolonged progression-free survival [120].

**Figure 2:**
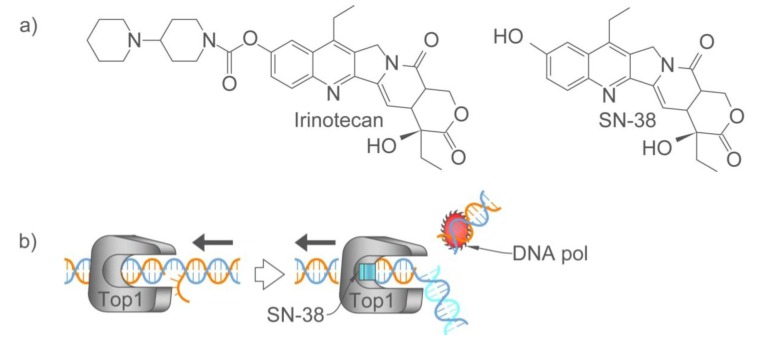
(**a**) Chemical structure of irinotecan and its metabolite, and (**b**) the mechanism of action of irinotecan, DNA topoisomerase I (Top1) cleaves and unwinds the DNA, as part of DNA replication and transcription. SN-38 forms a complex with DNA and Top1, inhibiting the religation of the DNA strand and blocking the movement of the DNA polymerase along the DNA. Consequently, a DNA double strand break ensues.

Another agent that is being used in combination with 5-FU/LV is irinotecan. Irinotecan (or CPT-11) is converted *in vivo* [121] to SN-38 (Figure 2a) [122] with the carboxylesterase-mediated removal of a bulky piperidino side chain [123]. Combining irinotecan with 5-FU/LV has increased response rate and prolonged time to progression and overall survival versus treatment with 5-FU/LV [124]. The target of irinotecan is DNA topoisomerase I (Top1), another enzyme involved in DNA metabolism. Top1 catalyzes DNA unwinding in an ATP-independent manner, a critical step in DNA replication [125] and transcription [126,127]. Top1 cleaves one of the DNA strands, facilitating the smooth rotation of the complementary strand around the first strand, and reseals the nick in the first strand [128,129]. The irinotecan metabolite SN-38 binds to the DNA-Top1 complex, inhibiting the religation of the nicked DNA strand (Figure 2b) [130]. The SN-38-DNA-Top1 complex can also impede the movement of DNA polymerase along the DNA strand and a double strand break ensues [131,132]. Irinotecan has been used in combination with 5-FU/LV (FOLFIRI), with a response rate among colorectal cancer patients of 49% [124].

#### 2.2.2. Who May and May not Benefit from Oxaliplatin and Irinotecan

Using oxaliplatin (in the FOLFOX regimen) and irinotecan (in the FOLFIRI regimen) have similar benefits in terms of response rate [133,134]. These two agents, however, have different adverse effects. Oxaliplatin causes an acute and transient neurotoxicity and a chronic, cumulative sensory neuropathy [135]. On the other hand, irinotecan can cause diarrhea in 16–24% of patients [136]. It is important to identify predictive parameters for response and toxicity that can be used to guide the choice between oxaliplatin and irinotecan.

**Scheme 4:**
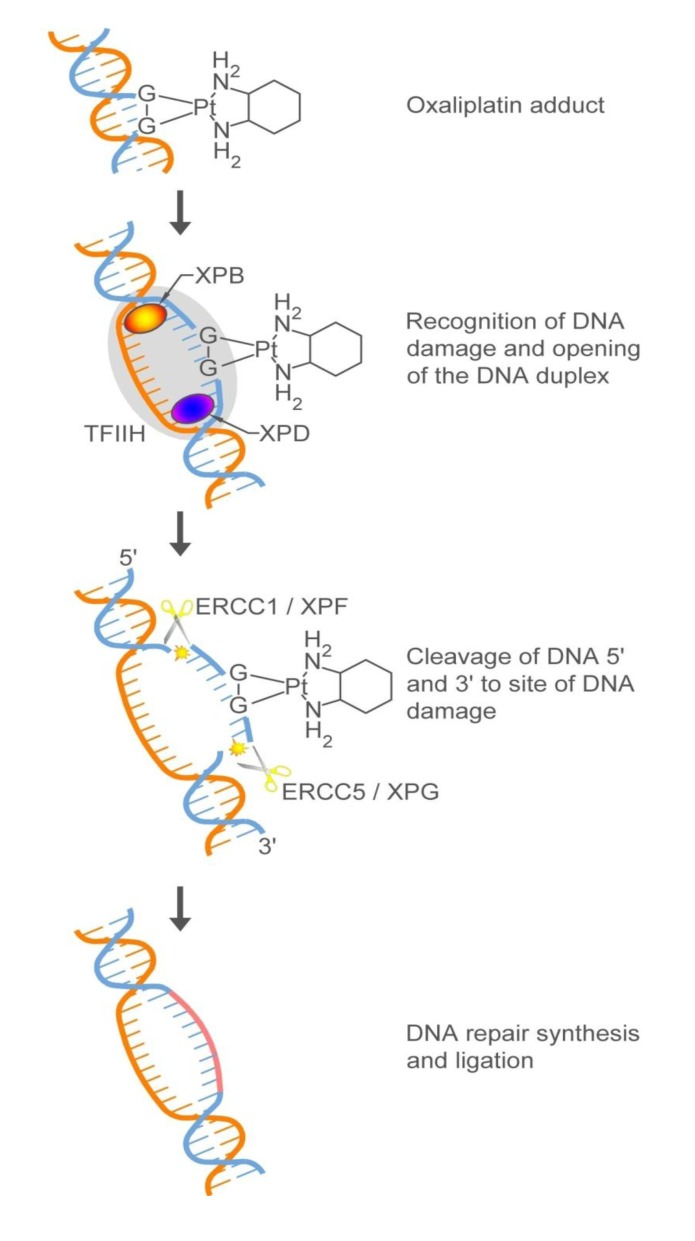
Schematic of the nucleotide excision repair pathway showing a subset of the proteins involved, especially those that have been implicated in response or resistance to oxaliplatin.

The response to oxaliplatin has been shown to be influenced by DNA repair capacity. The DNA adducts formed by oxaliplatin do not appear to be processed by the mismatch repair pathway [117] but may be removed by the nucleotide excision repair (NER) system (Scheme 4). At least 30 proteins [137] execute the different steps of NER that include recognition of DNA damage or structure distortion, separation of the DNA strands, DNA strand cleavage at the site of damage, and gap filling [138]. Polymorphisms have been identified in a subset of genes encoding for NER proteins [139], and a subset of these polymorphisms may be used to screen for oxaliplatin response or resistance.

ERCC2/XPD is part of a nine-subunit complex, TFIIH that is involved in the initial recognition of DNA damage [140]. ERCC2/XPD is one of two helicases in TFIIH that catalyzes the opening of the DNA duplex at the site of DNA damage [141]. It has been observed to be upregulated in response to oxaliplatin treatment *in vitro* and in the context of oxaliplatin resistance. A polymorphism, 13181T>G that results in a change from a lysine to a glutamine in codon 751(Lys751Gln) has been associated with decreased response (or increased resistance) to oxaliplatin [142,143,144,145]. However, the mechanism behind the effect of this *ERCC2* polymorphism on oxaliplatin response has not been elucidated.

ERCC1, in complex with another protein XPF, and ERCC5/XPG cleave the DNA 5' and 3', respectively, to the site of the DNA damage [146,147]. Patients with low *ERCC1* mRNA expression in their tumors survive longer in response to treatment with 5-FU/oxaliplatin [148] than patients with elevated *ERCC1* expression. Among the different polymorphisms that have been identified in the *ERCC1* gene, one has been associated with response to oxaliplatin—118C>T. Although this polymorphism does not result in an amino acid change, the resultant variant codon (AAT) is less frequently used than the common codon (118C) [149], possibly resulting in decreased ERCC1 expression [150], and presumably, less efficient NER. This can explain in part why patients that have the variant allele respond better to oxaliplatin-based treatment [150,151]. In the case of *ERCC5*, two polymorphisms, −763A>G and +25A>G, have been found to be associated with response to 5-FU/LV and oxaliplatin. Patients that had the -763GG had a significantly higher response rate than patients with either the AG or AA genotypes. On the other hand, the variant G allele at site 25 was associated with poorer response. These results, however, need further verification, especially since they were obtained from one clinical study of 83 patients [152].

A subset of patients has been shown to experience oxaliplatin-induced neurotoxicity despite a lack of response to the drug. Thus, withholding oxaliplatin from these patients can potentially spare them from the toxic effects of the drug without sacrificing therapeutic goals [153]. Platinum compounds can be detoxified with the attachment of a glutathione residue by glutathione S-transferases (GSTs) [154]. There are at least eight distinct GST classes; namely, alpha, kappa, mu, omega, pi, sigma, theta and zeta [155], which differ in substrate specificities [156]. The pi class (GST P1-1) is overexpressed in a number of cancers [157], including colon cancer [158,159,160]. GSTP1 overexpression has been associated with resistance to cisplatin [161,162]. Polymorphisms in the gene coding for GSTP1, *GSTP1*, have been identified, specifically in exon 5 (Ile105Val) and exon 6 (Ala114Val) [163]. Normal lung tissues from individuals with the Ile105Val polymorphism have reduced GST activity [156]. There are indications that patients with this polymorphism experience oxaliplatin-induced neurotoxicity earlier [164] or are more susceptible to more severe side-effects [165]. In one study, this same polymorphism was also predictive of response to FOLFOX-4. It has been proposed that as a result of reduced GST activity in patients with this genetic variant, oxaliplatin is detoxified at a lesser extent and is therefore more effective. The flip side of that is that is causes more toxicity [165]. On the other hand, in another study, the polymorphism was associated with a decreased risk of oxaliplatin-induced neurotoxicity [166]. In a meta-analysis, however, of 12 prospective trials and two retrospective clinical trials involving 2,191 participants, this polymorphism was not found to associate with oxaliplatin-induced neurotoxicity [167]. One limitation of this meta-analysis, however, is the analyses of trials that differ in specific chemotherapeutic regimens. In three trials where an association between the polymorphism and neurotoxicity has been observed [168], patients were treated with FOLFOX-4. Because of the conflicting results, however, on the association of the GSTP1 polymorphism with oxaliplatin toxicity, it is not currently recommended to screen patients for this genetic variant.

No genetic polymorphism has been identified that is associated with response to irinotecan. On the other hand, genetic variants have been identified that are associated with irinotecan toxicity. Irinotecan-induced diarrhea is attributed to the levels of the irinotecan metabolite SN-38 in the intestinal lumen [136]. SN-38 is glucuronidated by UDP-glucuronosyl transferase1A (UGT1A) to facilitate SN-38 elimination [169,170]. Low rates of glucuronidation have been associated with severity of irinotecan-induced diarrhea [171]. A *UGT1A1* allele, UGT1A7*3, codes for a protein sequence with three nonsynonymous single nucleotide polymorphisms; namely, Asn129Lys, Arg131Lys, Trp208Arg [172]. The protein that it codes for has a significantly lower catalytic activity than that coded for by the wild-type allele [170,173]. Consequently, at least in part, patients who are homozygous UGT1A7*3 have a 2.7 fold risk of experiencing diarrhea at the end of the treatment [174]. *UGT1A1* gene expression is inversely correlated to the number of TA repeats in its promoter TATA box [175]. The UGT1A1*28 allele has seven repeats in contrast to the six repeats in the wild type [176,177], and therefore, patients with this allele would have lower UGT1A activity as manifested by lower glucuronidation rates [178]. The number of UGT1A1*28 alleles (*i.e*., whether a patient is homozygous or heterozygous) is inversely correlated with the glucuronidated SN-38/SN-38 ratio [179]. Patients that are homozygous UGT1A1*28 have a significantly higher risk of experiencing diarrhea, hematologic and non-hematologic toxicities in response to irinotecan-based chemotherapy [174,179,180].

The UGT1A1*28 allele has been widely accepted as a predictor of irinotecan toxicity. Studies have been conducted on using information on the presence or absence of this allele to guide irinotecan dosing decisions [181]. The FOLFIRI regimen combines 5-FU/LV with 180 mg/m^2^ irinotecan [133]. However, results of a phase II trial indicate that there is advantage in terms of efficacy when a higher dose, 260 mg/m^2^, is administered [181,182]. Patients with the wild type *UGT1A1* allele (UGT1A1*1) can be given as much as 390 and 340 mg/m^2^ irinotecan (depending on whether a patient is homozygous or heterozygous, respectively, for the allele), in combination with 5-FU/LV [181,183]. These results demonstrate how the knowledge of a patient’s genotype can be used to guide drug dosing.

Currently, a patient’s genotype is not largely considered when choosing to administer oxaliplatin or irinotecan. Studies covered in this section of the review suggest that there may be benefit to screening for specific genetic variants to maximize efficacy and decrease toxicity in patients. Prospective clinical trials are necessary to test the hypothesis that genotype-guided decision making can increase response and decrease toxicity in patients treated with oxaliplatin and irinotecan.

## 3. Targeting New Mechanisms of Action: Targeting Cancer Cell-Specific Characteristics

A modern approach to cancer therapy involves use of chemical or biologic agents that interfere with a specific molecular target that has been shown to be involved in tumor growth and progression (referred to as targeted agents) [184]. We discuss below how the choice of targeted agents that are used in combination with 5-FU/LV can be personalized.

### 3.1. Personalizing Treatment with Antibodies against the Epidermal Growth Factor Receptor (EGFR)

#### 3.1.1. How Antibodies against EGFR Work

**Figure 3:**
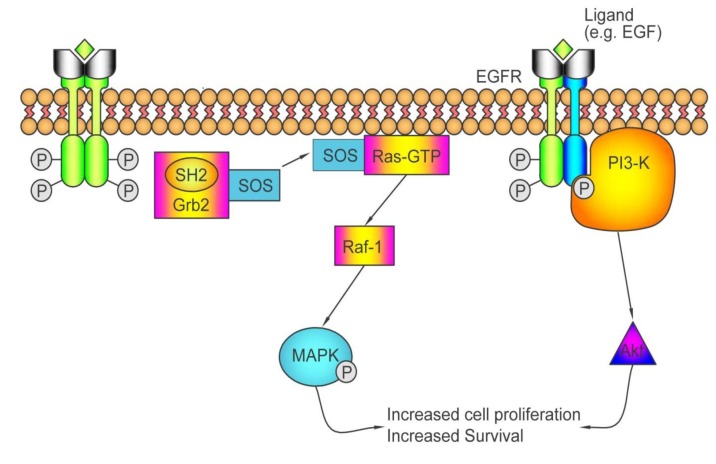
Activation of the EGFR pathway and the consequent engagement of the Ras/Raf/MAPK and PI3K/Akt pathways.

Higher expression of the epidermal growth factor receptor (EGFR) has been found in colorectal tumors of more advanced stage and poor differentiation, and those exhibiting vascular and lymphatic invasion [185]. Also, EGFR expression and tyrosine kinase activity are upregulated in metastatic colon cancer cells [186]. Consequent to binding to its ligand, which includes epidermal growth factor, EGFR phosphorylates itself and recruits a number of intracellular proteins containing Src homology 2 or phosphotyrosine binding domains [187]. A number of pathways are then engaged, including two that are involved in tumorigenesis; namely, the Ras/Raf/mitogen-activated protein kinase pathway (Ras/Raf/MAPK) and the phosphatidylinositol 3-kinase (PI3K)/Akt pathway [188] (Figure 3). The engagement of these two pathways promotes cell survival and proliferation. Mouse monoclonal antibodies raised against the EGFR (mAb 225IgG1 and mAb 528 IgG2a) block the binding of EGFR ligands and inhibit proliferation *in vitro* [189,190], tumor growth *in vivo* [191,192,193], and EGF-activated tyrosine kinase activity [194]. MAb 225IgG1 also induces receptor dimerization and downregulation [195]. This particular mouse monoclonal antibody has been chimerized to the human IgG1 constant region [196] and the chimera is now referred to as cetuximab (C225, Erbitux^®^, ImClone). Cetuximab has been as effective as mAb225 IgG1 *in vitro*, and more effective than mAb225 IgG1 in inhibiting tumor growth *in vivo* [197]. Cetuximab is immunogenic in about 5% of patients, however [198,199]. Thus, a fully human antibody (and not a human-mouse chimera) against EGFR, panitumumab [200] has been developed and has also been shown to inhibit tumor growth *in vivo*.

Given the upregulation of EGFR expression and activity in colorectal cancer, small molecule inhibitors of EGFR have also been developed and tested for colorectal cancer therapy. Unfortunately, no or minimal objective response (1% response rate) was observed in patients treated with the small molecule inhibitor gefitinib as a single agent [201,202] or in combination with capecitabine [203,204]. Furthermore, the addition of gefitinib to irinotecan did not seem to augment treatment efficacy [205]. Nevertheless, there is potential in combining an EGFR inhibitor with an EGFR antibody. In a clinical study of 50 patients, the combination of the EGFR inhibitor erlotinib with cetuximab was more effective than cetuximab alone [206].

#### 3.1.2. Who May and May not Benefit from Antibodies against EGFR

Lack of response to cetuximab or panitumumab-mediated blockade of the EGFR can be brought about by mutations that result in constitutive activation of the Ras/Raf/MAPK and/or PI3K/Akt pathways. The Ras proteins of the Ras/Raf/MAPK pathway bind the guanine nucleotides GTP and GDP, and have intrinsic GTPase activity [207]. They cycle between a GTP-bound active state and an inactive GDP-bound state [208]. GTPase activating proteins (GAPs) stimulate the GTPase activity of Ras proteins while guanine nucleotide exchange factors (GEFs) activate Ras by stimulating the release of GDP bound to Ras and exchange of GTP [209,210]. When EGFR binds to its ligand and the receptor dimerizes, a Ras GEF, Son of Sevenless (SOS), is recruited to the receptor, and Ras is subsequently activated [211]. The *K-ras* gene is one of three genes that code for one of four Ras proteins; namely, H-Ras, N-Ras, K-Ras4A, and K-Ras4B. Mutations in the *K-Ras* gene have been detected in 35–40% of colorectal cancers while mutations in genes encoding for other Ras proteins, *N-Ras* and *H-Ras* are only found in 1–3% of colorectal cancers [212,213]. Most of the mutations in the *K-Ras* gene have been detected in codon 12 [212], with the most frequent mutation being the substitution of glycine with aspartate (*i.e.*, G12D). The second most prevalent mutation (22.5% of samples tested) also changes codon 12—from glycine to valine (G12V). Twenty percent of *K-Ras* mutations are in codon 13, substituting an aspartate for the glycine (*i.e*., G13D) [213,214].

Clinical studies have shown that cetuximab and panitumumab are not effective in patients with a *K-Ras* mutation in codon 12 or 13 [215,216,217,218,219]. In light of this, it has now become standard to screen for mutations at these sites prior to prescribing cetuximab or panitumumab [216]. These mutations code for K-Ras proteins that have impaired GTPase activity. In addition, the GTPase activities of these mutant K-ras proteins are not stimulated by GAPs [209]. The mutant K-Ras proteins remain bound to GTP and are therefore, constitutively active [209,220]. Consequently, Ras downstream pathways are activated in a growth factor receptor-independent manner. In two large retrospective studies, however, it has been found that among patients with mutant *K-Ras*, patients with the *K-Ras G13D* mutation may benefit from cetuximab-containing chemotherapy (40–43% response rate with cetuximab versus 22% response without cetuximab) [221,222], even if their response is still lower than patients with wild-type *K-Ras* [222]. Patients with G13D respond better to cetuximab than patients with *K-Ras* codon 12 mutations [222]. This can be explained in part by *in vitro* results that showed that levels of activated Ras are lower in cells transfected with *K-RasG13D* than in cells transfected with *K-RasG12V* [223,224]. On the other hand, there are a number of *in vitro* results that show that cells possessing the *K-Ras G13D* mutation are resistant to cetuximab [225,226]. It has even been observed that cells that have been induced to become cetuximab-resistant by continuously exposing them to cetuximab have acquired either *K-Ras G12V* or *K-Ras G13D* mutations [227]. Moreover, in a large retrospective study of patients treated with panitumumab, the outcome in patients with the *G13D* mutation was not better than other *KRas* mutations. This study confirmed that only patients with wild-type *K-Ras* benefit from panitumumab-containing chemotherapy [228]. It is possible that the benefit seen among patients with *K-Ras G13D* mutants in response to cetuximab but not panitumumab is brought about in part by the ability of cetuximab to induce antibody-dependent cellular cytotoxicity (ADCC) [229]. Cetuximab is an IgG1 antibody while panitumumab is an IgG2 antibody. IgG1 antibodies have a high affinity for the Fc gamma receptors (FcγRs) found on the surfaces of immune effector cells, e.g. natural killer (NK) lymphocytes and macrophages. IgG1but not IgG2 antibodies interact with FcγRIIIa that is expressed by NK cells. Thus, tumors coated by an IgG1 antibody, like cetuximab (and not panitumumab) can be recognized by NK cells and be susceptible to NK-mediated cytolysis, a mechanism that is referred to as antibody-dependent cellular cytotoxicity (ADCC) [230]. Given the retrospective nature of the studies that showed that patients with *K-RasG13D* may potentially benefit from cetuximab, it is recommended that this finding be verified in prospective trials. In another study, it has been shown that patients with this mutation can also benefit from an antibody against vascular endothelial growth factor, bevacizumab (discussed below) [231]. *N-Ras* mutations, although not as prevalent as *K-Ras* mutations in colorectal cancer, may also be predictive of poor response among wild-type *K-Ras* patients [232].

Administering cetuximab or panitumumab only to patients that have wild-type *K-Ras* keeps potentially resistant patients from experiencing undue side effects. However, even among wild-type *K-Ras* patients, up to 65% of them do not respond to chemotherapy incorporating anti-EGFR antibodies [216,221]. Thus, the search for more markers predictive of response/resistance to EGFR antibodies, in addition to *K-Ras*, continues.

Active GTP-bound Ras activates the Raf serine/threonine kinases (B-Raf, C-Raf, and A-Raf), resulting in the activation of MAPKs. Mutations in the *B-Raf* gene have been found in 5–17% of colorectal cancers. Approximately 90% of the *B-Raf* mutations codes for a substitution of glutamic acid for the wild-type valine in residue 600 (*i.e.*, V600E), resulting in a constitutively active protein [233]. In two clinical studies, only 2 out of 26 patients with mutant *B-Raf* responded to cetuximab or panitumumab, even if these patients had wild type *K-Ras* [232,234]. It has been shown *in vitro* that resistance to cetuximab in colorectal cancer cell lines with mutant *B-Raf* can be reversed by combining cetuximab with a B-Raf kinase inhibitor sorafenib [234]. A recent case report describes the benefits of this strategy in treating a patient who has a *B-Raf* mutation [235]. Combining an EGFR inhibitor with a B-Raf kinase inhibitor is worth exploring in clinical studies [236]. The next most common *B-Raf* mutation in colorectal cancer is D594G (<1% of colorectal cancers) [232,237]. In a study of 370 wild-type *K-Ras* colorectal tumor samples, this mutation was detected in one sample. This sample came from a patient that responded to cetuximab [232].

The EGFR does not only activate the Ras/Raf/MAPK pathway but also the PI3K/Akt pathway. Similar to the impact of activating mutations in the players in the Ras/Raf/MAPK signaling cascade, mutations resulting in the activation in the PI3K/Akt pathway are expected to impart resistance to EGFR antibodies. EGFR, among other growth factor receptors, activates Class I_A_ PI3Ks. The class I­_A_ PI3Ks are heterodimers of a regulatory subunit (of which the most studied is p85α or p85) and a 110kDa catalytic subunit [238]. There are three isoforms of the catalytic subunit; namely, 110α, 110β, and 110δ [238]. Mutations in the gene coding for the p110 α subunit, *PIK3CA,* have been detected in up to 32% of colorectal cancers analyzed [239,240,241]. The most prevalent mutations lie on sequences that are in the helical and kinase domains of the protein, specifically in exons 9 and 20 [242]. The H1047R, E542K and E545K mutations, all detected in colorectal cancers, code for mutant proteins with higher lipid kinase activity than the wild-type protein [241,243]. These mutations result in Akt activation and cellular transformation [243]. In a clinical study involving 110 patients, none of the patients with the abovementioned *PIK3CA* gene mutations respond to panitumumab or cetuximab [244]. In another study that included 370 wild-type *K-Ras* patients, patients with mutant *PIK3CA*, particularly H1047R, had a significantly lower response rate than patients with wild-type *PIK3CA* [118]. The PI3K/Akt pathway is negatively regulated by phosphatase and tensin homolog (PTEN). Thus, PTEN downregulation can result in activation of the PI3K pathway. Majority of patients that have a dramatic reduction/loss of PTEN expression in their tumors do not respond to cetuximab [244,245].

Mutations in the *K-Ras*, *B-Raf*, and *PIK3CA* and loss of PTEN expression are predictive of resistance to EGFR antibodies. However, no parameter has been established to help predict which patients will gain the maximum benefit from these agents. *EGFR* gene copy number or *EGFR* amplification, as assessed by fluorescence or chromogenic *in situ* hybridization, is associated with response to cetuximab-involved chemotherapy [218,246,247,248,249]. One of the hurdles in the use of EGFR gene amplification as a positive predictor for response, however, is the conflicting results at least in part due to differences in methodology. Assessing *EGFR* gene copy number by RT-PCR in 34 patients did not demonstrate an association between *EGFR* copy number and response [250]. In the case of the use of FISH for *EGFR* amplification analysis, there has been difficulty in standardizing the protocol. Even established pathology labs that were given the same samples assessed *EGFR* gene amplification status differently. This issue needs to be addressed before *EGFR* gene copy number testing can be used to identify cetuximab- or panitumumab-responsive patients [251].

There are indications that sensitivity to EGFR antibodies among patients with wild-type *K-Ras* is positively associated with the mRNA expression of the EGFR ligands epiregulin and amphiregulin in tumor samples [219,252]. Downregulating amphiregulin or epiregulin *in vitro* decreases response to cetuximab [253]. The mechanism, however, behind the association between epiregulin/amphiregulin expression with cetuximab sensitivity remains to be elucidated.

The decision to incorporate EGFR antibodies in a colon cancer chemotherapeutic regimen also involves the choice between using cetuximab or panitumumab. Although both cetuximab and panitumumab are antibodies against EGFR, their binding sites are not identical. This can explain in part why patients can respond to panitumumab even after progressing under cetuximab [254,255]. Panitumumab can still bind to an EGFR mutant S492R to which cetuximab cannot bind to. This particular EGFR mutant was detected in tumors of patients that progressed under cetuximab [255]. It has been found that although the panitumumab epitope overlaps with the binding site of cetuximab, there are still distinctions between the binding sites of these two EGFR antibodies [256]. It remains to be seen how the differences in their binding specificities can be used to guide treatment decisions as to which EGFR antibody to use.

### 3.2. Personalizing Treatment with Inhibitors of Vascular Endothelial Growth Factor

Neovascularization, the formation of new blood vessels, is necessary for rapid tumor growth [257,258]. The extent of neovascularization, as manifested by the number of microvessels in the tumor, has been correlated with the disease stage [259,260], and recurrence rate in colorectal cancer [260,261]. Vascular endothelial factor (VEGF or VEGF-A) is the predominant regulator of angiogenesis in colon cancer. VEGF expression has been correlated with microvessel density in colon tumors [259,261], with expression being higher in tissue from patients with more advanced disease and metastases [259,262]. Other pro-angiogenic factors that have structural similarities to VEGF-A are VEGF-C, and VEGF-D [263] and placenta growth factor (PlGF) [264] Expression of PlGF has also been correlated with colorectal cancer progression [265,266]. VEGF-A is recognized by two receptor tyrosine kinases, VEGFR-1 (Flt-1) and VEGFR-2 (KDR or Flk-1) [267]. VEGF-C and -D bind to VEGFR-2 and VEGFR-3 [263], and PlGF binds only to VEGFR-1 [268].

Different strategies have been developed to inhibit VEGF-induced angiogenesis. One is through the use of antibodies specific against VEGF. Bevacizumab is the humanized form [269] of a mouse antibody against human VEGF that has been shown to inhibit tumor growth and reduce microvessel density *in vivo* [270]. In colorectal cancer patients, injecting bevacizumab has been shown to reduce tumor vascularity [271] and tumor perfusion [272]. The use of bevacizumab in combination with other chemotherapeutic agents (5-FU, LV, oxaliplatin, irinotecan) has improved survival [273,274,275,276,277,278].

Another strategy to inhibit angiogenesis is not only to inhibit VEGF but also to trap other pro-angiogenic factors. Aflibercept (VEGF Trap) has been developed by fusing sequences encoding for the binding domains of VEGFR-1 and VEGFR-2 with the Fc region of human IgG1 antibody. This fusion protein therefore can bind both VEGF-A and placental growth factor, and inhibit angiogenesis. Similar to bevacizumab, aflibercept can significantly improve survival, when administered in combination with FOLFIRI [279].

#### 3.2.1. Who May and May not Benefit from Inhibitors of VEGF

The addition of bevacizumab or aflibercept to chemotherapeutic regimen, although potentially beneficial, significantly increases the occurrence of adverse events [278,279]. It is prudent, therefore, to identify patients who can gain the maximum benefit with the least toxicity.

A subset of *VEGF* genetic polymorphisms has been associated with variability in VEGF protein levels [280]. Polymorphisms that have been associated with lower VEGF production may predict for sensitivity to bevacizumab-containing chemotherapy. Patients with the -634G>C (also reported as +405G>C) whether in heterozygous or homozygous form have a significantly higher response rate to FOLFIRI+bevacizumab than patients who are homozygous *634G* [281]. The variant has been independently associated with decreased VEGF production by peripheral blood mononuclear cells that have been treated with lipopolysaccharide [282]. The value of screening for this polymorphism remains to be established given that in a different study, the variant has been associated with higher serum VEGF levels in healthy Japanese individuals [283]. Patients with the −152G>A and 1154G>A *VEGF* polymorphisms survived longer than patients with the respective homozygous wild-type alleles [281]. The 1154 G/G genotype is more common in patients who do not respond to chemotherapy combining bevacizumab with 5-FU (oral or infusional) and irinotecan than in patients who respond [284]. Peripheral blood mononuclear cells from healthy individuals who are homozygous *−1154A* produce significantly less VEGF than those from individuals who are homozygous for the *−1154G* allele [285,286]. It is conceivable then that patients with the 1154 G>A polymorphism also produce less VEGF and therefore, respond better and survive longer in response to bevacizumab-based therapy. It is important to note that the predictive value of a particular *VEGF* polymorphism on response to bevacizumab is influenced by what agents are co-administered with the VEGF antibody. Among patients treated with FOLFOXIRI + bevacizumab, no association has been found between a *VEGF* polymorphism and response [287]. In patients treated with a regimen involving capecitabine (the oral prodrug of 5-FU), oxaliplatin, and bevacizumab, a single *VEGF* polymorphism is not predictive of outcome. However, the combination of the *VEGF 405C* allele with a thymidylate synthase polymorphism (*TYMS* 3G) is predictive of progression-free survival [288]. Another parameter that needs to be controlled in predicting response from *VEGF* sequence is the choice of sample. A study on colorectal cancer tissue did not show a correlation between the *1154G>A* allele and tumor VEGF mRNA expression [289]. On the other hand, when the presence of this polymorphism was assessed in blood samples, the polymorphism has been shown to be correlated with VEGF production by PBMC’s and survival outcome in response to FOLFIRI+ bevacizumab.

The number of circulating endothelial cells (CECs) in patients is a potential predictor of response to chemotherapy containing bevacizumab. CECs are believed to be shed from blood vessel walls as a result of vascular damage, although their biology is still not well understood. Nevertheless, they have been found to be more numerous in metastatic CRC patients than healthy subjects before treatment. Patients that have lower baseline CECs have a better survival outcome in response to bevacizumab- containing chemotherapy than those with higher CEC counts [290,291]. Among the different CEC phenotypes that express different markers, the percentage of CECs expressing the chemokine receptor CXCR4 (CXCR4-CECs) is more predictive of survival outcome [292] in response to bevacizumab than those of CECs expressing other markers; namely, VEGFR-1, VEGFR-2, and the angiopoietin receptor Tie2. Patients that have lower percentage of CXCR4-CECs (distinguishing cut-off: 20% CXCR4-positive) survived significantly longer than those with higher values. The interaction between CXCR4 and its ligand, stromal cell-derived factor 1 alpha (SDF-1 alpha), may play a role in tumor neovascularization in a VEGF-independent manner [293]. This can explain in part why high number of CXCR4-expressing CECs is predictive of poor response to bevacizumab. The use of CECs to guide chemotherapeutic decisions regarding bevacizumab still needs to be optimized. Conflicting results as to its predictive power [291,294] may be in part due to differences in methodologies in isolating them. Given that more recent work has shown that a particular subtype of CECs, the CXCR4-CECs, is more predictive of benefit than other CEC subtypes [292], methods have to be optimized to accurately quantitate CXCR4-CECs.

Other biomarkers for predicting and monitoring response and resistance to bevacizumab are being developed given the technical difficulty in detecting and monitoring CECs [295,296]. The most logical biomarker that has been explored is VEGF itself. The appropriate methodology, however, to measure VEGF remains under study. Measurement of total VEGF can include VEGF molecules that are actually bound to the VEGF antibody, and are therefore biologically inactive. On the other hand, assessment of free, and presumably, biologically active VEGF may be more indicative of the efficacy of the therapy. Free VEGF can be measured by passing serum samples through a column that removes all IgGs, including antibody that is bound to VEGF [297]. Total VEGF levels increase in response to bevacizumab [297,298,299]. Nevertheless, levels of free VEGF (*i.e.*, unbound to antibody) decrease in response to bevacizumab [287,297,300]. Thus, amount of free and not total VEGF may be a better biomarker of response. Tissue samples may not be the best choice in assessing response to bevacizumab. When VEGF expression is assessed from tissue samples, no association is found between VEGF levels and response or survival outcomes [301].

### 3.3. Personalizing Treatment with Multi-Kinase Inhibitors

**Figure 4:**
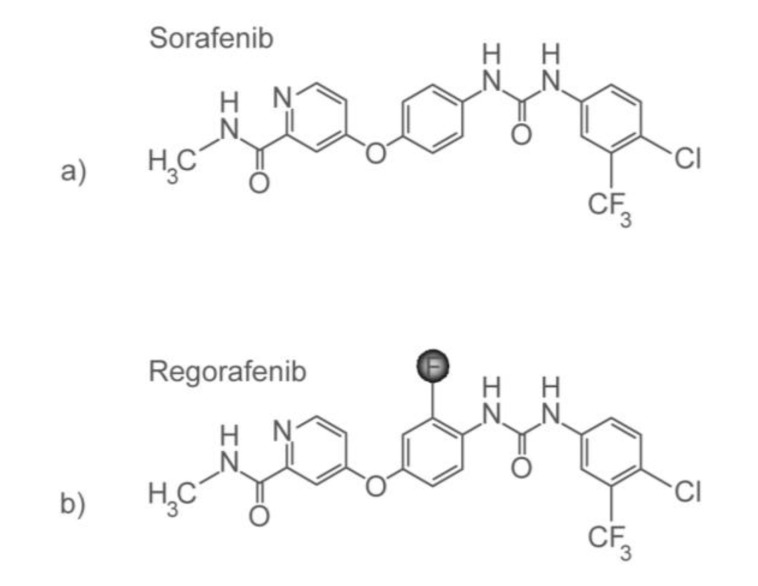
Chemical structures of (**a**) sorafenib and (**b**) regorafenib.

Resistance to inhibitors of EGFR and VEGF can be brought about by activation of alternate pathways that increase proliferation and promote survival in an EGFR- and VEGF-independent manner. A strategy to address this resistance is to design inhibitors against the signaling kinases involved in these alternate pathways. A mutation in the *B-Raf* gene leads to activation of the MAPK pathway [302], independent of EGFR activation. Blocking Raf-induced activation of the pathway has been shown to inhibit transformation *in vitro* and tumor progression *in vivo* [303], prompting the search for Raf inhibitors. In a high-throughput screen, 3-thienyl urea was identified as a reversible Raf kinase inhibitor. This discovery was made amidst findings from other groups that show that ureas may inhibit kinases [304]. Chemical modifications on the lead compound, 3-thienyl urea, were done to increase its activity, eventually resulting in the synthesis of the derivative, BAY 43-9006 (sorafenib), a bis-aryl urea [305] [Figure 4(a)]. Sorafenib inhibits the MAPK pathway in colorectal cancer cell lines (among other cancer cell types) that have *K-Ras* or *B-Raf* mutations [306].

Raf may not only have a role in cell proliferation but also in angiogenesis. Raf can be activated by basic fibroblast growth factor (bFGF) and VEGF, and inhibit endothelial cell apoptosis [307]. Injecting mice with nanoparticles conjugated to the mutant *B-Raf* gene to block endogenous endothelial Raf activity, inhibits angiogenesis [308]. Sorafenib has not only been shown to inhibit transformation and tumor growth but also angiogenesis. It blocks the tyrosine kinase activity of the pro-angiogenic VEGFR-1, -2, and -3 and platelet-derived growth factor receptor-β [309]. In Phase I studies, 42% of colorectal cancer patients treated with sorafenib experience disease stabilization [310]. Given its Raf kinase inhibitory activity, combining sorafenib with EGFR inhibitors is being considered. It is synergistic with cetuximab in inhibition of transformation (as assessed by a soft agar assay) *in vitro* [311]. The benefit of the use of sorafenib with CRC remains under investigation, whether it is used as a single-agent or in combination with other chemotherapeutics [312,313]. The impact of sorafenib is more on disease stabilization, even in combination with irinotecan or oxaliplatin [314,315].

Regorafenib, like sorafenib, is also a bi-aryl urea compound. The only difference between the two is a fluorine atom in regorafenib (Figure 4b). Through mechanisms that remain undiscovered, this single change results in a distinct kinase inhibition profile. In addition to the kinases mentioned above that are inhibited by sorafenib, regorafenib inhibits Tie2—the receptor of another proangiogenic factor angiopoietin. Regorafenib inhibits cell proliferation *in vitro* and inhibits angiogenesis *in vivo* of colorectal cancer cells. When regorafenib treatment of mice is terminated, a slow regrowth of Colo-205 (colorectal cancer) xenografts has been observed [316]. In a Phase I study of colorectal cancer patients, disease stabilization is seen in 19 out of 27 patients (70%), and tumor perfusion is decreased in most patients [317]. In a Phase III study, regorafenib prolonged overall survival in comparison to giving best supportive care only, in patients with metastatic colorectal cancer whose disease progressed with currently approved standard chemotherapeutic regimen. Regorafenib treatment results mostly in disease stabilization, rather than tumor shrinkage [318].

The use of multi-kinase inhibitors in colorectal cancer is yet to be personalized. These inhibitors have been recently developed and their combination with other colon cancer chemotherapeutics is still the subject of exploration. It would be a challenge to identify patients who are susceptible or resistant by screening for polymorphism in one to three genes, given that these agents target multiple kinases. Strategies to personalize treatment with these inhibitors can involve monitoring drug levels and monitoring response more closely by assessing kinase inhibition and tumor angiogenesis.

### 3.4. Personalizing Treatment with an Inhibitor Specific for Mutant B-Raf

Patients who have a *B-Raf* mutation have been shown to have a poorer outcome than patients that have wild-type *B-Raf* [319,320,321]. Specifically, in colorectal cancer, more than 95% of the *B-Raf* mutations that have been found in colorectal cancer are V600E mutations [232,237]. A selective mutant *B-Raf* kinase inhibitor, vemurafenib (RG7204, PLX4032, RO5185426, Plexxicon/Genentech), has been developed [322]. It is currently being used for the treatment of metastatic or unresectable BRaf^V600^ melanoma. It has been shown that vemurafenib is also effective against a subset of colorectal cancer cell lines that had the *B-Raf* V600E mutation [323]. Unfortunately, when it was tested in the clinic with 19 evaluable patients, only one patient exhibited a response [324]. Similar to the results with *K-Ras* inhibitor, mutations in PIK3CA and reduction in PTEN promote resistance of *B-Raf* V600E colorectal cancer cells to vemurafenib [325]. Thus, patients that have mutant *B-Raf* , mutant *PIK3CA,* and PTEN downregulation may not benefit from vemurafenib alone. On the other hand, EGFR levels have been shown to affect response to vemurafenib, with low EGFR-expressing cells being susceptible to vemurafenib. EGFR activation [326,327] and consequent Akt phosphorylation [325,326] have been observed in cells treated with the *B-Raf* inhibitor, correlating with resistance. It is conceivable that EGFR upregulation does not contribute to a poor response if cells have low levels of EGFR. Combining an EGFR inhibitor with a B-Raf inhibitor has been shown to inhibit tumor growth in mice [326,327]. A related strategy that has promise is the combination of a B-Raf inhibitor with an Akt inhibitor [325]. More recently, it has also been shown that there is benefit in combining a B-Raf inhibitor with a PI3K/mTOR inhibitor given the observed activation of the PI3K/mTOR pathway in B-Raf inhibitor-treated cells [328]. In the clinic, the inhibition of *B-Raf* (by using the multi-kinase inhibitor sorafenib) and the inhibition of EGFR appeared to prolong survival [235]. A step that can be taken towards personalizing treatment with inhibitors specific for mutant *B-Raf* is to assess *PIK3CA* status and PTEN and EGFR expression. If the patient that has mutant *B-Raf* V600 also has wild-type *PIK3CA* mutation and PTEN is not downregulated, vemurafenib can be used. The assessment of EGFR expression can guide the decision on whether to combine vemurafenib with an EGFR inhibitor.

### 3.5. Personalizing Treatment in View of Colorectal Cancer Stem Cells (Co-CSC) and Circulating Tumor Cells (CTC)

One important task in our efforts to personalize treatment of CRC patients is to detect chemoresistance and/or disease recurrence and treat patients appropriately. When dissociated tumor cells from patient samples are treated with 5-FU, although the viability of the bulk population decreases, it has been found that the surviving population is enriched with a subset of cancer cells referred to as cancer stem cells (CSC) [329,330]. Cancer stem cells make up less than 10% of a tumor. Yet, they have been shown to be the cell subset that actually have the capability to form tumors, self-renew, and differentiate to recapitulate the characteristics of the primary tumor [331]. Because of how important it is to isolate, study, and eventually target CSC, efforts have been made to identify CSC-specific markers. In the case of colorectal cancer, putative markers include CD133 [332,333,334], EpCAM (epithelial adhesion molecule), CD44, CD166 [335], aldehyde dehydrogenase 1 (ALDH1) [336], CD26 [329], CD24, CD29, and Lgr5 [337].

It is proposed that one of the reasons behind treatment failure in patients is the resistance of cancer stem cells to chemotherapeutics. Although chemotherapeutics may affect the non-stem cell population, the surviving stem cell population can self-renew. Clinically, this can be observed as disease progression or recurrence. CD133+ cells that were purified from colon cancer samples have been found to be more resistant to 5-FU, oxaliplatin and the pro-apoptotic protein TRAIL than CD133- cells [334]. CD26+ cells are resistant to 5-FU and oxaliplatin [329] and CD44+CD166+ cells are resistant to irinotecan [330]. CD133 positivity in patients’ primary tumors has been found to be associated with a poorer response to chemotherapy [338,339]. Thus, one way to personalize CRC treatment is to assess the predominance of cancer stem cells in patients’ primary tumors. This can help guide the choice of chemotherapeutic. The choice of sample, however, is critical. To predict chemotherapeutic response from CD133 expression, for instance, primary tumor samples need to be used. In metastatic tissue, both CD133+ and CD133- cells possess stem cell characteristics [340]. Thus, the extent of CD133 positivity in metastases may not be indicative of chemotherapeutic response/resistance.

Another cancer cell subset that may be used to predict response to chemotherapy is the circulating tumor cell (CTC). The presence of circulating tumor cells, *i.e*., tumor cells in the blood, was first described in 1869. Since then, researchers have devised methods to detect them more efficiently [341]. In 2004, the US Food and Drug Administration approved the use of an immunomagnetic CTC selection and enumeration system in the clinic. Based on the results of a clinical trial involving 430 metastatic colorectal cancer patients, the threshold count has been set to three CTC’s in 7.5 mL blood [342]. A patient is predicted to have poor prognosis if three or more CTC’s are detected in their blood sample. A number of clinical studies have shown that not only the baseline CTC count may be important but also the trend of the CTC count in response to treatment [343]. It has been shown that patients whose CTC counts decreased from an unfavorable number (*i.e.*, above the threshold) to a favorable one had similar survival outcomes than those patients who maintained a favorable CTC count throughout chemotherapy [342,344]. the increase in CTCs in patients during chemotherapy may be an early predictor of resistance. Furthermore, patients with high baseline CTC who continue to have high CTC even after treatment have poor survival outcomes [345]. The trend in the patient’s CTC counts may be as effective as radiographic imaging in showing a lack of response to the treatment [346]. The number of CTC's in a patient's blood after different durations of treatment is predictive of survival outcomes. Given this, it has been proposed that CTC counts can help guide decisions regarding whether and how long of a treatment break can be given to the patient [342].

## 4. Conclusions

**Figure 5:**
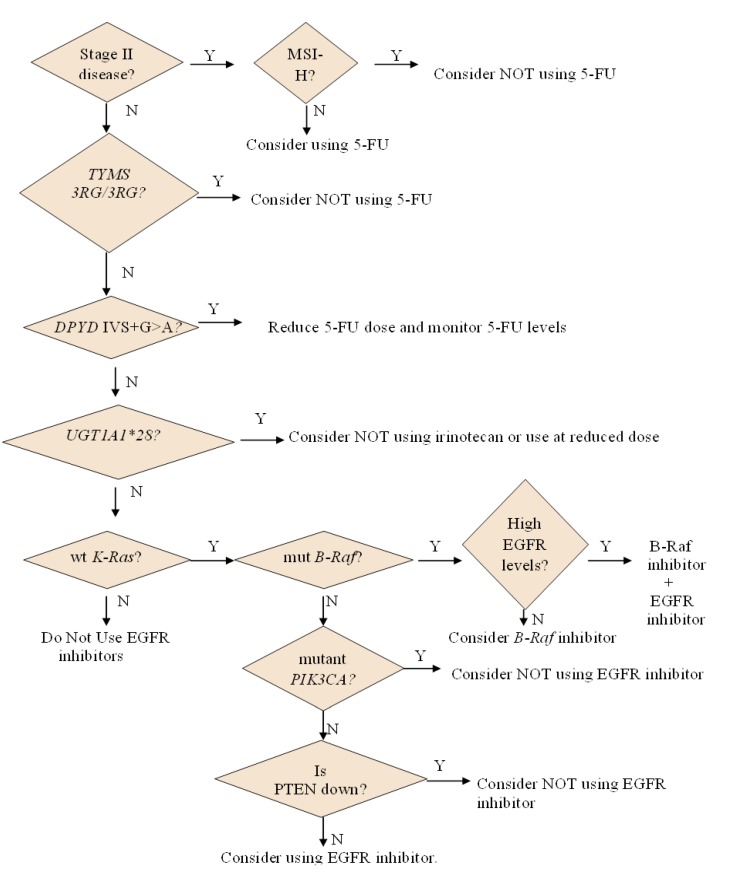
A decision-making flowchart that illustrates how the presence/absence of specific genetic mutations and polymorphisms discussed in this review article can be used to guide therapeutic decisions.

In this review, we discussed how the use of old and new colon cancer chemotherapeutics is being personalized. Genotyping an individual to detect polymorphisms in the *TYMS*, *MTHFR*, *DPYD*, *UGT1A*, *ERCC1*, *K-Ras*, *B-Raf*, and *PIK3CA*, can help guide the choice of chemotherapeutic agent (Figure 5). As large scale genomic analyses are performed more often, the number of genes (e.g., *N-Ras*, *Aurora kinase*, *CDK8*) and genetic changes (e.g., gene fusions) that can be used in the decision-making process is expected to increase [347]. Aside from genetic markers, however, there are other predictive parameters that can be used. PTEN and EGFR protein expression, *EGFR* and *TYMS* gene copy number, and number of circulating endothelial cells are additional markers that can be used to predict response. Personalizing treatment does not only involve choosing what chemotherapeutic to administer but also adjusting the dose of the drug to maximize benefit and minimize toxicity. Pharmacokinetic monitoring and dose adjustment of 5-FU has been shown to increase response and reduce toxicity. In addition, irinotecan doses can be optimized based on the knowledge of what *UGT1A1* allele a patient has.

The real-life benefits of years of research to personalize colon cancer chemotherapeutics are increasingly being seen. In the clinic, a patient can have his tumor screened for specific polymorphisms and mutations that would predict what drugs they will respond to, and what drugs they may react adversely to. In particular, results of screening for the *K-Ras* mutation determine whether a patient will even be given an EGFR inhibitor. Once patients start 5-FU based chemotherapy, 5-FU pharmacokinetic monitoring can be performed and 5-FU doses adjusted based on actual drug levels. There is, however, more to be done. Because important clinical decisions are made based on laboratory tests, the reliability of these tests must be assured. An example of how different results from two genetic testing laboratories may have affected management of disease has been reported in a case report by Lamparella *et al.* [348]. In their experience, two reference laboratories obtained two different findings on the *K-Ras* mutation status of a patient. Given that EGFR antibodies are withheld from patients with mutant *K-Ras*, it is understandable how the conflicting results affected treatment decisions. It is imperative that standards for ensuring the reliability of results of genetic testing be laid out, to promote maximum clinical benefit to the patient.

A critical area of research is the understanding of the mechanisms behind chemotherapeutic failure. Possible reasons behind drug failure include tumor chemoresistance or suboptimal drug levels. There are no methodologies in place to ensure that the molecular targets of the chemotherapeutics are really being inhibited in the patient. Finally, as can be gleaned from the response rates with current chemotherapeutic regimens and the median overall survival of 2 years for metastatic colorectal cancer, one of the greatest challenges remains the development of more effective but safe pharmaceuticals. As more information is gained about an individual’s normal and tumor genotype, especially with the optimization of whole genome sequencing, potentially, we can match the drug that will best fit the individual.
